# Characterization and genomic analysis of *Lactiplantibacillus plantarum* LP8 as a probiotic candidate for medical applications

**DOI:** 10.1016/j.dib.2025.111555

**Published:** 2025-04-11

**Authors:** Nirusna Jehma, Nattarika Chaichana, Jirasa Boonsan, Kamonnut Singkhamanan, Monwadee Wonglapsuwan, Rattanaruji Pomwised, Sarunyou Chusri, Komwit Surachat

**Affiliations:** a Department of Biomedical Sciences and Biomedical Engineering, Faculty of Medicine, Prince of Songkla University, Hat Yai, Songkhla 90110, Thailand; b Division of Biological Science, Faculty of Science, Prince of Songkla University, Hat Yai, Songkhla, 90110, Thailand; c Division of Infectious Diseases, Department of Internal Medicine, Faculty of Medicine, Prince of Songkla University, Songkhla 90110, Thailand; d Translational Medicine Research Center, Faculty of Medicine, Prince of Songkla University, Hat Yai, Songkhla 90110, Thailand

**Keywords:** *Lactiplantibacillus plantarum*, Whole genome sequencing, Probiotics, Draft genome sequence, Safety assessment, Antimicrobial resistance gene

## Abstract

This study presents the whole genome sequencing (WGS) and functional analysis of *Lactiplantibacillus plantarum* LP8, a promising probiotic strain, is presented in this research. The genome comprises a 3.23 Mbp circular chromosome and three plasmids including plasmid1_LP8 (58,764 bp), plasmid2_LP8 (45,003 bp), and plasmid3_LP8 (7985 bp). Functional annotation identified 3151 coding sequences (CDSs) mapped to 209 RAST subsystems, alongside biosynthesis gene clusters encoding Plantaricin J and other secondary metabolites such as RiPP-like peptides and terpenes. Safety analysis revealed no acquired antimicrobial resistance gene (AMR) or virulence genes, with only intrinsic resistance to certain antibiotics. In vitro analysis confirmed its sensitivity to antibiotics such as ampicillin, erythromycin, and tetracycline, and its hemolytic activity displayed an α-hemoly-sis pattern. These findings confirm *L. plantarum* LP8 as a safeand effective probiotic candidate with significant potential for antimicrobial and biotechnological applications.

Specifications Table

**Table:** 

Subject	Genomics
Specific subject area	Microbial genomics
Type of data	Genomic sequencing data, predicted genes, and genome annotation
Data collection	Whole genome sequencing of short-read with Illumina MiSeq platform long-read with the Oxford Nanopore Technologies (ONT) system, following de novo genomic assembly.
Data source location	Institution: Department of Biomedical Sciences and Biomedical Engineering, Faculty of Medicine, Prince of Songkla University, Hat Yai, Songkhla, Thailand
Data accessibility	Data are publicly available at NCBI GenBank to the BioProject database under the identifier PRJNA1196694 (https://www.ncbi.nlm.nih.gov/bioproject/?term=PRJNA1196694), and the associated BioSample can be accessed using the number SAMN45628971 (https://www.ncbi.nlm.nih.gov/biosample/?term=SAMN45628971).

## 1 Value of the Data

• The genomic characterization of *L. plantarum* LP8, isolated from fermented shrimp, provides novel insights into its genetic adaptations and functional potential as a probiotic strain, which serves as a valuable reference for comparative genomic studies of *L. plantarum* strains from diverse fermented food sources.
• The identification of biosynthetic gene clusters involved in bacteriocin and secondary metabolite production suggests potential applications in antimicrobial development. These findings may contribute to the discovery of novel bioactive compounds for food preservation, pathogen inhibition, and gut microbiome modulation.
• The genomic data confirm the absence of acquired antimicrobial resistance (AMR) and virulence genes, supporting safety profile of the strain for probiotic applications.
• The presence of functional genes linked to stress tolerance, carbohydrate metabolism, and biofilm formation highlights the adaptability of the strain that makes it a promising candidate for fermented food enhancement, gut health improvement, and biotechnological applications.
• This dataset provides a foundation for future studies, including metabolomic, transcriptomic, and in vivo probiotic efficacy assessments, contributing to the development of functional probiotic strains for medical and food industry applications.

## 2 Background

Lactic acid bacteria (LAB) are widely recognized for their role in food fermentation and potential health benefits as probiotics [1]. Among these, *Lactiplantibacillus plantarum* is a highly versatile and resilient species, widely recognized as one of the most common bacteria found in fermented foods, probiotics, dairy products, and beverages. *L. plantarum* has been extensively studied for its safety, functionality, and genome characteristics. Moreover, *L. plantarum* enhances the functional properties of fermented foods by producing bioactive compounds such as exopolysaccharides (EPS), γ-aminobutyric acid, riboflavin, folic acid, and vitamin B12. It is widely used in food processing and preservation due to its ability to produce potent bacteriocins and organic acids, offering broad-spectrum antimicrobial activity against various pathogens [2]. Therefore, this research explores the genotypic traits, functional properties, and safety of *L. plantarum* LP8, isolated from Thai-style fermented shrimp in Hat Yai, Songkhla, Thailand, with a focus on genome classification, safety, and drug resistance analysis with safety assessment by in vitro analysis.

## 3 Data Description

**Fig. 1:**
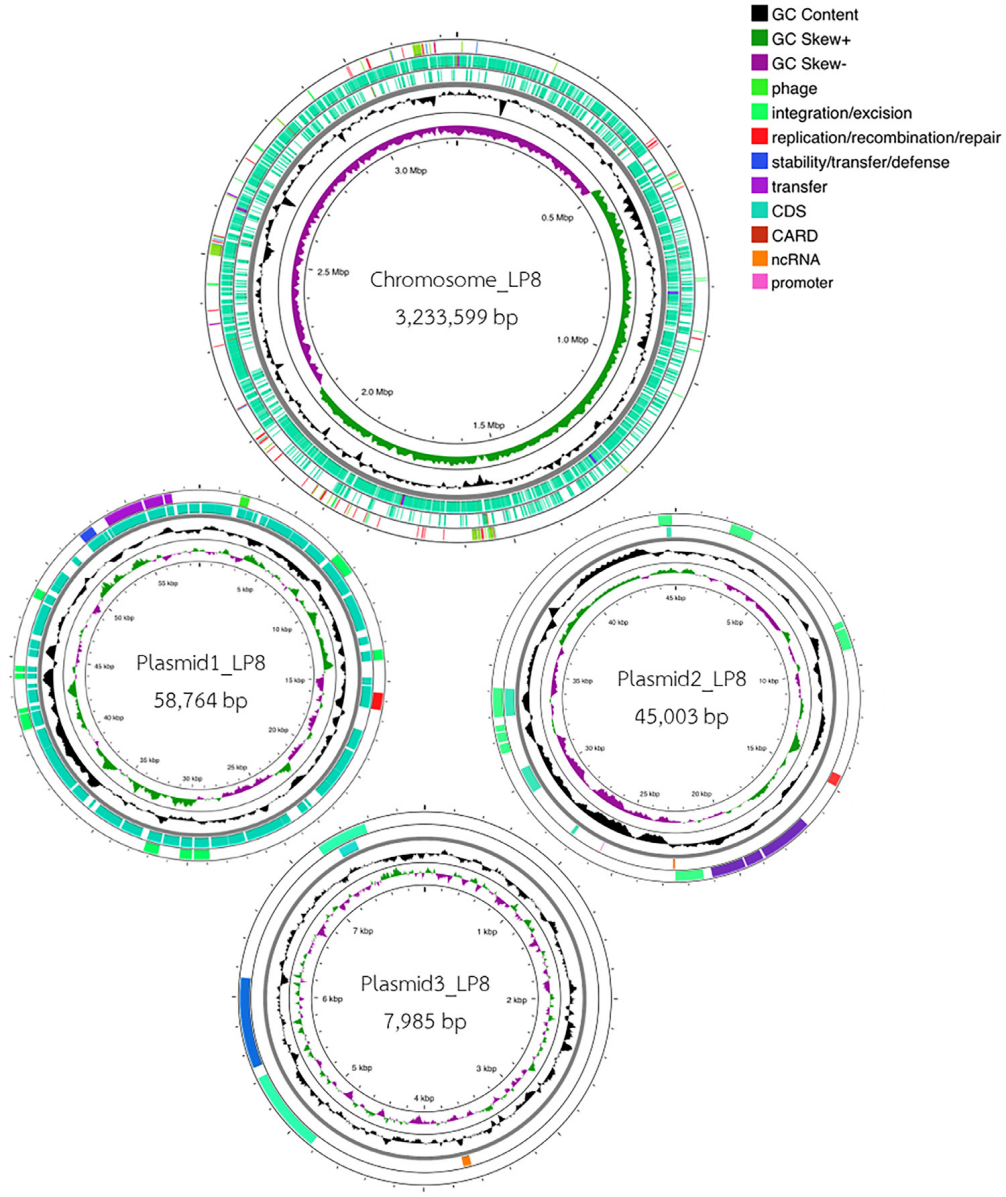
Circular genome visualization of *Lactiplantibacillus plantarum* LP8. The circular chromosome (3,233,599 bp) and three plasmids: plasmid1_LP8 (58,764 bp), plasmid2_LP8 (45,003 bp), and plasmid3_LP8 (7,985 bp).

This report presents the whole genome sequencing (WGS) data of *Lactiplantibacillus plantarum* LP8, highlighting its genome features, functional annotation, antimicrobial resistance, virulence factors for safety evaluation, and metabolite biosynthesis gene clusters. The assembled genome sequence of LP8 consists of a 3.23 Mbp circular chromosome and three circular plasmids named plasmid1_LP8 (58,764 bp), plasmid2_LP8 (45,003 bp), plasmid3_LP8 (7,985 bp) as visualized in Fig. 1. Moreover, functional annotation identified 3,151 coding sequences (CDSs) mapped to 209 RAST subsystems in the chromosome. For plasmid annotations, 73, 41, and 14 CDSs were identified in pLP8-1, pLP8-2, and pLP8-3, respectively. These were assigned to 5 and 1 RAST subsystems for pLP8-1 and pLP8-2, respectively, while no RAST subsystems were identified for pLP8-3. The genome also contained 16 rRNA genes, 70 tRNA genes, and 1 tmRNA (Table S1). The plasmid comparison presented in Fig. S1 exhibits the genetic similarity between the plasmids of *L. plantarum* LP8 and those from various other strains. Sequence alignments revealed that plasmid1_LP8 shares significant similarity with the plasmid of *L. plantarum* strain pc-26 (p.pc-2601, Accession number: CP023302.1), exhibiting 79% coverage and 99.9% sequence identity. Similarly, plasmid2_LP8 aligns with the plasmid named unnamed3 from *L. plantarum* strain ZG308 (Accession number: CP183363.1), with 55% coverage and 96.27% identity. Moreover, plasmid3_LP8 shows strong alignment with the plasmid unnamed5 from *L. plantarum* strain E2 (Accession number: CP110247.1), with 73% coverage and 97.19% identity. These comparisons suggest that the plasmids of LP8 are genetically similar to those found in other *L. plantarum* strains, indicating the presence of conserved regions responsible for essential functions such as replication, metabolic processes, and resistance mechanisms. The high sequence identity across all three plasmids further suggests that these plasmids are evolutionarily conserved [3,4]. To assess its safety and probiotic potential, the ProbioMin server was used to evaluate functional properties [5]. The safety analysis revealed no acquired antibiotic resistance genes (AMRs), except for the *clpL* gene, which is linked to disinfectant resistance rather than antibiotic resistance. Moreover, no virulence factor-associated genes were detected in the Virulence Factor Database (VFDB), while the presence of two prophage regions (PPRS) was classified as low risk. However, two genes, *walR* and *cspR*, were identified with 84.25% and 84.61% identity, respectively. These genes are essential for probiotic functionality rather than pathogenicity. The *walR* regulates cell wall metabolism, ensuring structural integrity and adaptability in harsh environments such as the gastrointestinal (GI) tract. The *cspR* encodes a cold shock protein that helps the strain tolerate temperature fluctuations and stress, enhancing viability during storage and transit. Given that both genes are chromosomally located and not associated with mobile genetic elements, their potential for horizontal gene transfer (HGT) is minimal.

**Fig. 2:**
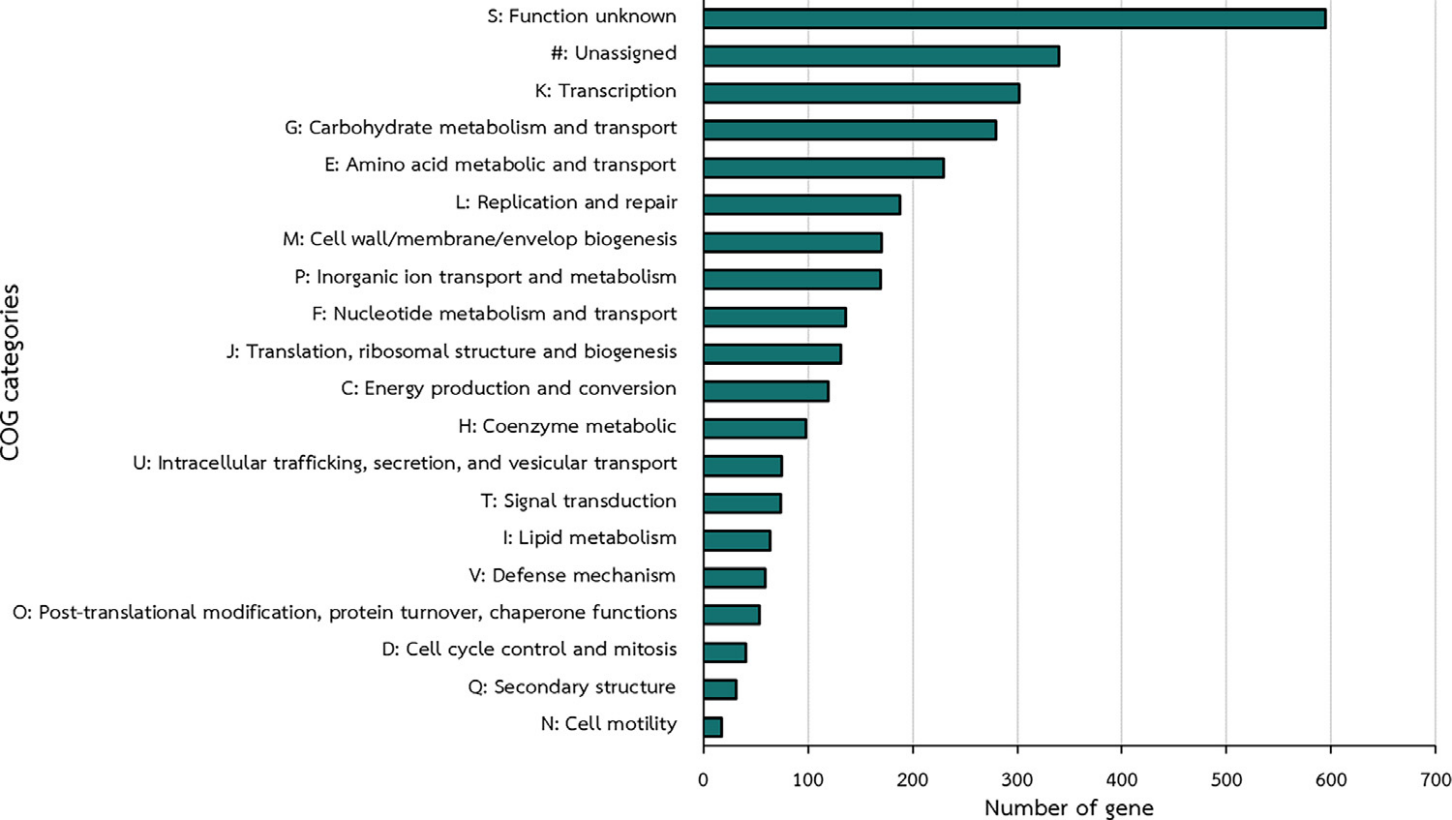
Distribution of coding sequences (CDSs) of *L. plantarum* LP8 across Cluster of Orthologous Groups (COG) functional categories.

Only 2,238 in silico-predicted proteins of LP8 were assigned to Clusters of Orthologous Genes (COGs). Notably, the most abundant categories highlight its genetic and metabolic adaptability, with a significant portion of genes in the function unknown and unassigned categories, suggesting potential for novel discoveries [6]. Key functions include transcription, carbohydrate and amino acid metabolism, and replication, reflecting the strong regulatory and metabolic systems essential to this strain for probiotic activity (Fig. 2). The biosynthetic potential and functional adaptability of LP8 were further explored through the identification of biosynthetic gene clusters (BCGs) involved in the production of bioactive compounds that are listed in Table S2. The Plantaricin J cluster encodes a bacteriocin with potent antimicrobial activity, while RiPP-like and cyclic-lactone autoinducer clusters may play a role in secondary metabolite production and microbial communication [7].

The antimicrobial susceptibility profile of LP8 was evaluated through laboratory experiments, which demonstrated sensitivity to ampicillin, erythromycin, and tetracycline, intermediate susceptibility to gentamicin, and intrinsic resistance to vancomycin, streptomycin, chloramphenicol, kanamycin, and clindamycin (Table S3). Notably, genomic analysis did not detect any acquired antimicrobial resistance (AMR) genes, reinforcing the idea that its resistance is intrinsic rather than acquired [8]. In addition, hemolytic activity displayed an alpha (α) hemolysis pattern, indicating partial red blood cell breakdown, characterized by a greenish-brown discoloration resulting from partial hemoglobin reduction. This pattern is not associated with pathogenicity, as alpha hemolysis is commonly observed in non-pathogenic lactic acid bacteria. It occurs due to hydrogen peroxide production by this bacterial group, which induces oxidative damage to red blood cells. Many probiotic strains, including various *L. plantarum* species, naturally exhibit alpha hemolysis without posing any risks to human health [9,10].

## 4 Experimental Design, Materials, and Methods

### 4.1 DNA extraction and whole genome sequencing (WGS)

The genomic DNA (gDNA) of *L. plantarum* LP8 was extracted using the ZymoBIOMICS™ DNA Miniprep Kit (Zymo Research, Irvine, CA, USA) and further purified with the DNeasy extraction kit (QIAGEN, Hilden, Germany) following the manufacturer's protocols. The concentration and purity of the gDNA were assessed using a Qubit Fluorometer (Thermo Fisher Scientific, Inc.), and its integrity was verified by agarose gel electrophoresis. The extracted and purified gDNA was then prepared for whole-genome sequencing (WGS) on both short-read by MGISEQ-2000 platform with 150-bp paired-end reads and long-read sequencing platform using the Oxford Nanopore Technologies (ONT) system [11]. The gDNA library for ONT was constructed using the Rapid Barcoding Kit 24 V14 (SQK-RAK114.24, Oxford Nanopore Technologies, Oxford, UK) according to the manufacturer’s instructions. The prepared library was loaded onto an R.10.4.1 flow cell and sequenced using the MinION Mk1C sequencer (Oxford Nanopore Technologies) following standard ONT protocols.

### 4.2 Genome assembly and functional annotation

The sequence was analyzed using Bactopia v3.0, an automated pipeline designed specifically for bacterial genome assembly and annotation. The genome was annotated using the Prokaryotic Genome Annotation System (Prokka v1.14.6) and the Rapid Annotations using the Subsystems Technology (RAST) webserver v1.073 (both accessed on November 18, 2024). Moreover, Cluster of Orthologous Groups (COG) annotations were performed using eggNOG-mapper v2 based on the eggNOG database. The genome was subsequently visualized using the Proksee server (accessed on December 05, 2024) [12]. Plasmid comparison was performed using Easyfig software.

### 4.3 Antimicrobial resistance and virulence factor-associated gene prediction

For the safety assessment of LP8, PathogenFinder v1.1 (accessed on December 05, 2024) was utilized to assess the pathogenicity in human hosts. Antibiotic resistance genes (ARGs) and virulence factors were detected using the Comprehensive Antibiotic Resistance Database (CARD v6.0.3), ResFinder v4.6.0, AMRFinderPlus v4.0.15, and the Virulence Factor Database (VFDB, BLASTN v2.8.1+) (all accessed on December 05, 2024) with an identity and coverage threshold set at 80%.

### 4.4 Bacteriocin-encoding gene and biosynthesis gene cluster (BCG) identifications

Bacteriocin-encoding genes in the genome were identified using Bagel4 v1.2 (accessed on December 06, 2024). Moreover, biosynthesis gene clusters (BCGs) of secondary metabolites were analyzed with antiSMASH v7.1.0 (accessed on December 06, 2024).

### 4.5 Antibacterial susceptibility test

The antimicrobial susceptibility profile of LP8 was examined using the agar disk diffusion method as previously described [13]. The antimicrobials used in this research are listed in Table S3, following the CLSI 2020 guidelines.

### 4.6 Hemolytic activity assay

Hemolytic activity of LP8 was performed on a blood agar plate containing 5% (v/v) of blood as previously described [14]. Hemolytic activities were classified as Beta (β) hemolysis with a clear of complete lysis, alpha (α) hemolysis with greenish-brown discoloration due to hemoglobin reduction, and gamma (γ) hemolysis showing no lysis.

## Limitations

Not applicable.

## Ethics Statement

This research did not involve any human subjects or animal experiments. No ethical approval was required.

## Credit Author Statement

**Nirusna Jehma**: Writing – review & editing, Writing – original draft, Validation, Software, Methodology, Investigation. **Nattarika Chaichana**: Writing – review & editing, Writing – original draft, Validation, Software, Methodology, Investigation. **Jirasa Boonsan**: Software, Methodology, Investigation. **Kamonnut Singkhamanan**: Writing – review & editing, Writing – original draft, Project administration, Methodology, Investigation, Conceptualization. **Monwadee Wonglapsuwan**: Writing – review & editing, Methodology, Investigation, Conceptualization. **Rattanaruji Pomwised**: Writing – review & editing, Validation, Methodology, Investigation, Conceptualization. **Sarunyou Chusri**: Writing – review & editing, Validation, Methodology, Investigation, Funding acquisition, Conceptualization. **Komwit Surachat**: Writing – review & editing, Writing – original draft, Validation, Supervision, Software, Resources, Project administration, Methodology, Investigation, Funding acquisition, Conceptualization.

## Data Availability

NCBILactiplantibacillus plantarum LP8 (Original data).

## Appendix Supplementary materials

Image, application 1
