# Supplementary material for: Characterization and genomic analysis of Lactiplantibacillus plantarum LP8 as a probiotic candidate for medical applications
Source: Data Brief. 2025 Apr 11;60:111555. doi: 10.1016/j.dib.2025.111555 (PMC12076797; doi:10.1016/j.dib.2025.111555)
Supplement: Supplementary file 1 [file mmc1.docx]

**Table S1** Genomic information of *Lactiplantibacillus plantarum* LP8.

| **Attribute** | **Chromosome** | **Plasmid-1** | **Plasmid-2** | **Plasmid-3** |
| --- | --- | --- | --- | --- |
| **Genome size (bp)** | 3,233,599 | 58,764 | 45,003 | 7,985 |
| **G+C content (%)** | 44.6 | 40.2 | 38.5 | 36.24 |
| **Number of CDS** | 3,151 | 73 | 41 | 14 |
| **tRNA** | 71 | 0 | 0 | 0 |
| **rRNA** | 90 | 0 | 0 | 0 |
| **tmRNA** | 1 | 0 | 0 | 0 |
| **ncRNA** | 0 | 0 | 1 | 1 |
| **RAST subsystems** | 209 | 5 | 1 | 0 |

**Table S2** Antibacterial susceptibility profile of LP8.

| Antimicrobial agent | Inhibition zone (mm) |
| --- | --- |
| Ampicillin (10 µg) | 26.00 ± 2.02 (S) |
| Vancomycin (30 µg) | 0.00 ± 0.00 (R) |
| Erythromycin (15 µg) | 32.00 ± 0.50 (S) |
| Chloramphenicol (30 µg) | 0.00 ± 0.00 (R) |
| Clindamycin (2 µg) | 20.00 ± 0.58 (S) |
| Streptomycin (10 µg) | 0.00 ± 0.00 (R) |
| Gentamycin (10 µg) | 9.00 ± 0.29 (I) |
| Kanamycin (30 µg) | 0.00 ± 0.00 (R) |
| Tetracycline | 26.00 ± 0.76 (S) |

**Table S3** The identification of bacteriocin and secondary metabolite-biosynthetic gene clusters (BCGs).

| Type | Compound name | Start | End |
| --- | --- | --- | --- |
| Bacteriocin | Plantaricin J | 109,370 | 138,865 |
| Secondary metabolite | RiPP-like | 111,952 | 124,102 |
|  | Cyclic-lactone-autoinducer | 568,244 | 588,949 |
|  | Terpene | 840,900 | 861,781 |
|  | T3PKS | 1,876,063 | 1,917,232 |


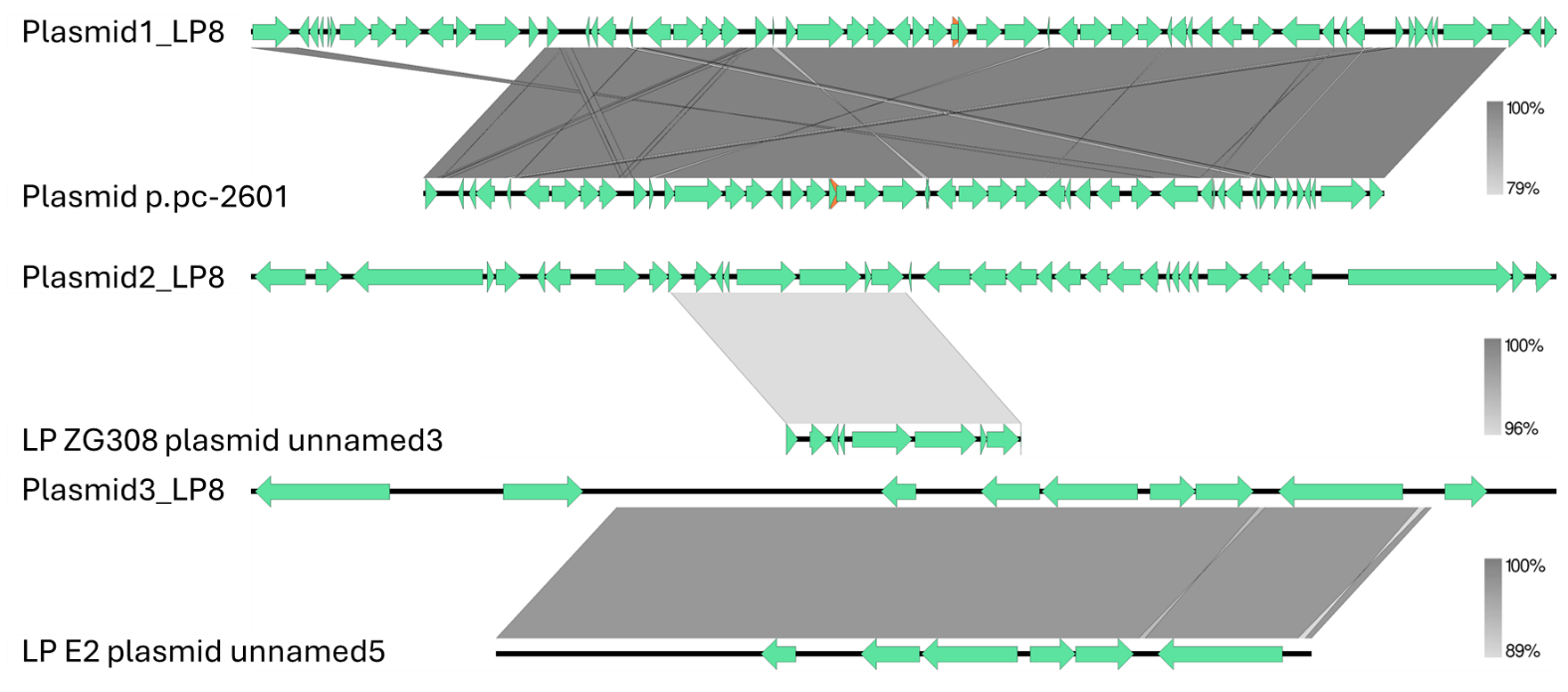


**Fig. S1** Plasmid comparison between *L. plantarum* LP8 and plasmids from other strains. The alignment of plasmid1_LP8 with the p.pc-2601 plasmid (Accession: CP023302.1), plasmid2_LP8 with the*L. plantarum* strain ZG308 plasmid unnamed3 (Accession: CP183363.1), and plasmid3_LP8 with the *L. plantarum* strain E2 plasmid unnamed5 (Accession: CP110247.1) are shown. The shaded grey areas represent regions of sequence homology between the compared plasmids.
